# HIFα independent mechanisms in renal carcinoma cells modulate divergent outcomes in fibronectin assembly mediated by hypoxia and CoCl_2_

**DOI:** 10.1038/s41598-020-75756-5

**Published:** 2020-10-29

**Authors:** Carina Magdaleno, Leah Dixon, Narendiran Rajasekaran, Archana Varadaraj

**Affiliations:** Department of Chemistry and Biochemistry, Northern Arizona University, Building 36, Room 430, PO Box 5698, Flagstaff, AZ 86004 USA

**Keywords:** Renal cell carcinoma, Cell migration

## Abstract

Fibronectin (FN) is a core matrix protein that assembles to form a dynamic cellular scaffold, frequently perturbed during oncogenic transformation. Tumor hypoxia, characterized by low oxygen concentrations in the microenvironment of most solid tumors has been shown to accelerate FN assembly in fibroblasts and cancer-associated fibroblasts, cell types that produce abundant amounts of FN protein. Nevertheless, FN matrix regulation in epithelial cancer cells during hypoxia remains less well defined. In this study we investigate the assembly of the FN matrix during hypoxia in renal cancer epithelial cells, the cells of origin of renal cell carcinoma (RCC). We show that hypoxia (1% O_2_) specifically increases matrix disassembly and increases migratory propensity in renal cancer cells. However, HIFα stabilization using hypoxia mimetics, does not recapitulate the effect of hypoxia on FN matrix reorganization or cell migration. Using a combination of knockdown and inhibitor-based approaches, our work characterizes the signaling events that mediate these two disparate changes on the matrix and explores its functional significance on chemotactic cell migration. Our study systematically reexamines the role of hypoxia mimetics as experimental substitutes for hypoxia and provides new findings on HIFα stabilization and the FN matrix in the context of renal cancer.

## Introduction

Clear cell renal cell carcinoma (ccRCC), accounts for 70–75% of the kidney cancer cases and is a rare metastatic cancer with a tendency to metastasize in an organ-specific manner^1,2^.

Metastasis is a multi-step cascade contributed by alterations in the amount and assembly of extracellular matrix (ECM) proteins, influencing invasion/migratory properties at the invasive front of a tumor for tumor dissemination^3,4^. Although stromal fibroblasts, myofibroblasts and cancer-associated fibroblasts (CAFs) are main players in the abundant secretion and assembly of ECM proteins^5–7^, the role of the epithelial cancer cell ECM in cancer is less well defined.

Fibronectin (FN) is a primordial ECM glycoprotein required for the assembly of other ECM proteins such as fibrillin^8^ and collagen^9^. Cell associated FN localizes in the cytoplasm as FN dimers and at the apical cell surface as FN fibrils. Upon binding of dimeric FN to Integrin α5β1 receptors at the cell surface, the integrin receptor is activated, creating cytoskeletal tension and force on the focal adhesion complex. This force leads to exposure of buried cryptic sites within the receptor-associated FN protein, allowing binding of more FN dimers, and assembly of a polymerized FN scaffold by a process called fibrillogenesis^10–12^. Studies carried out in several cancer types have demonstrated that polymerized FN (sFN) significantly decreases tumor growth, metastasis and angiogenesis^13,14^. Thus FN functions appear to vary depending on the polymerized state of the protein.

Tumor hypoxia, defined by low oxygen concentrations (approximately 1%) in most solid tumors^15^, is a key contributor of the tumor microenvironment (TME) that promotes the survival and adaptation of stromal and cancer cells to aid cancer progression^16^. During hypoxia, FN in fibroblasts align into unidirectional fibrils by a mechanism that is dependent on the Hypoxia inducible factor (HIF-1α)^17^. Increased ECM alignment has also been observed in cancer-associated fibroblasts (CAFs) compared to normal tissues^5,18,19^. However, in normal renal epithelial cells, hypoxia exposure increases the abundance of FN but not the formation of FN fibrils^20^. While the assembly state of FN in the tumor stroma guides tumor progression, the role of fibrillogenesis in epithelial cancer cell migration, response to hypoxia, or mechanisms of fibrillogenesis as a contributing factor to ccRCC tumor progression has not been evaluated.

Prolyl hydroxylase (PHD) inhibitors are commonly used hypoxia mimetics in establishing experimental hypoxia. Since PHD inhibition stabilizes HIFα, an indicator of hypoxia receipt in the cells, several research questions have been addressed using hypoxia mimetics interchangeably with hypoxia in in vitro and in vivo studies.

In this study, we investigate the role of hypoxia (1% O_2_) and the hypoxia mimetic CoCl_2_ on FN fibril assembly or fibrillogenesis. Our results in ccRCC cells reveal that hypoxia mimetics (and PHD2 inhibition) exhibit distinct effects on FN matrix organization compared to hypoxia in cells. We show that treatment with CoCl_2_ increases the assembly of FN into fibrils by a Rho-dependent and HIFα-independent mechanism. Using small molecule inhibitors and shRNA-based approaches we demonstrate that fibril assembly in response to CoCl_2_ does not involve the classical integrin α5 activation but requires the combined participation of integrins α4 and α5. Interestingly, treatment with another hypoxia mimetic DMOG or the knockdown of PHD2 does not increase FN fibril assembly nor does it recapitulate the disassembly of the FN matrix we observe when cells are exposed to hypoxia (1% O_2_). Thus our studies point to critical differences between hypoxia and HIFα stabilization approaches on the cancer epithelial cell FN matrix. Functionally, our data reveals a key difference in the migratory propensity in renal cells exposed to hypoxia compared to treatment with hypoxia mimetics and provides relevant findings on the role of hypoxia in driving renal cancer progression. This study sheds light on approaches using hypoxia mimetics in the context of matrix reorganization, influencing our scientific conclusions on cancer cell phenotype and function.

## Results

### FN matrix assembly decreases in response to 1% O_2_ or hypoxia but increases in the presence of the hypoxia mimetic CoCl_2_

**Figure 1:**
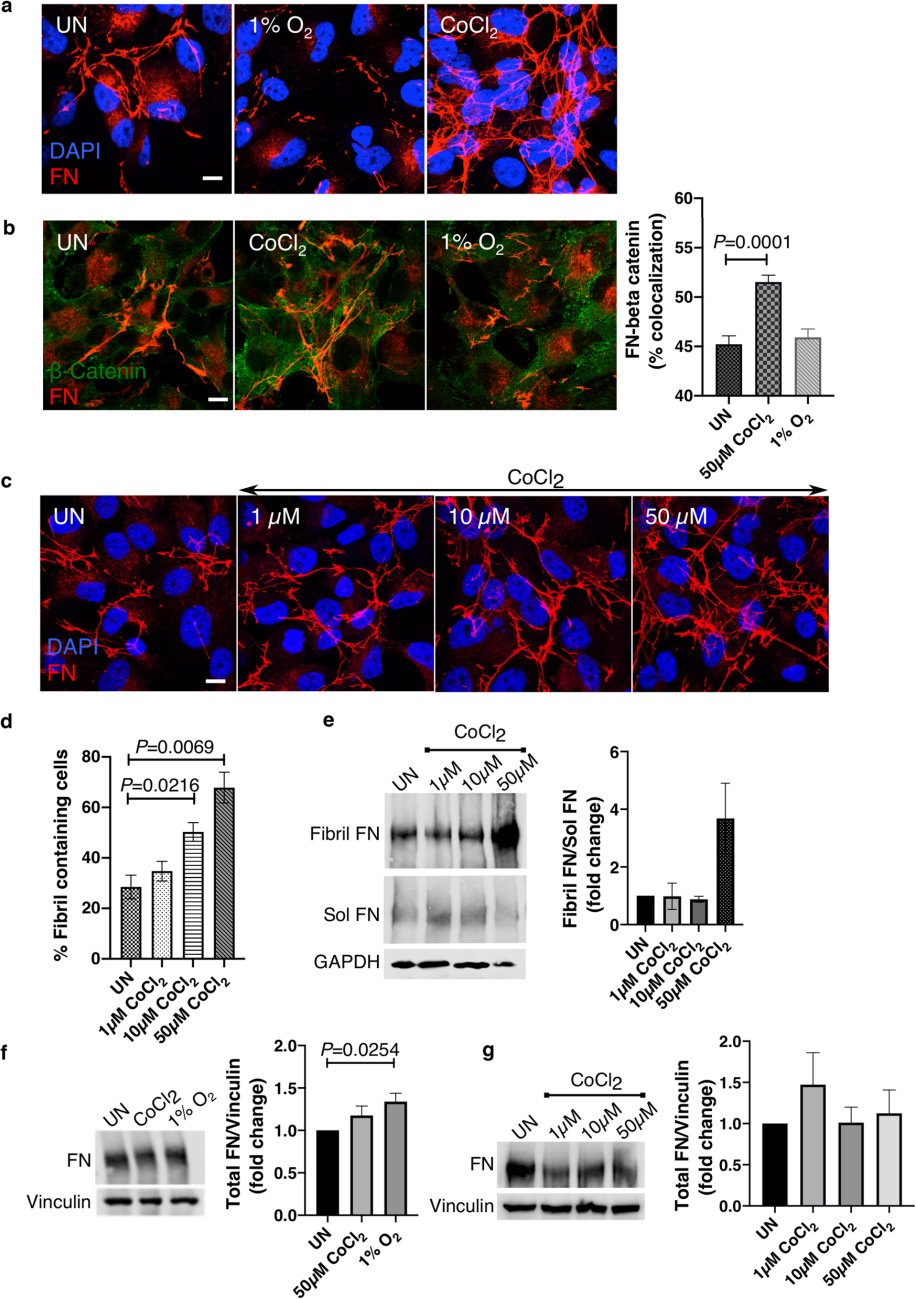
Fibril assembly decreases during hypoxia (1% O_2_) but is increased in CoCl_2_ treated Caki-1 cells. (**a**) Caki-1 cells were cultured in 21% O_2_ (UN), exposed to 1% O_2_ or treated with 50 µM CoCl_2_ for 18 h and immunostained for FN (red) and counterstained with the nuclear stain DAPI (blue). (**b**) Caki-1 cells treated as in (**a**) were immunostained for FN (red) and β-catenin (green). Each image is a single 1 µm z-slice. Bar graph on the right is the mean % colocalization (n = 90), ± SEM of the two proteins. Colocalization was analyzed from ROIs drawn at the membrane localization of β-catenin. Actual *P* values were calculated using the unpaired Student’s t-test. (**c**) Caki-1 cells were untreated or treated with 1 µM, 10 µM or 50 µM CoCl_2_ for 18 h and immunostained as in (**a**) Scale bar = 10 µm. Images in (**a**) and (**c**) are maximum intensity projections that includes all pixel values in each layer throughout the z-axis of each cell (**d**) Cells treated as described in (**c**) were divided into ‘fibril containing’ and ‘not containing’ and the % of ‘fibril containing’ cells were plotted as a bar graph as shown. A total of 100 cells were counted for each condition per experiment. Bar graph is an average of three independent experiments (n = 3), ± SEM. Actual *P* values were calculated using the unpaired Student’s t-test. (**e**) Cells treated with 1 µM, 10 µM or 50 µM CoCl_2_ for 18 h were lysed and cell lysates fractionated using deoxycholate to separate fibril FN and soluble FN. Quantification on the right shows the ratio of fibril versus soluble FN fractions, normalized to loading control GAPDH, plotted as mean ± SEM (n = 2). (**f**) Total cell lysates were lysed in SDS buffer to solubilize total FN pools (fibril and soluble combined) and immunoblotted against FN. Vinculin is used as the loading control. Quantification of the right indicates total FN levels normalized to vinculin plotted as mean ± SEM from three independent trials. Statistical significance and actual *P* values were determined using the unpaired Student’s t-test. (**g**) Total FN levels immunoblotted as in (**f**) with quantification on the right plotted as mean ± SEM of three independent trials.

To investigate the role of hypoxia on the FN matrix in renal cancer epithelial cells, we exposed Caki-1 cells to 1% O_2_ for 18 h and visualized FN by immunostaining. We observed a noticeable decrease in FN fibrils in response to hypoxia as confirmed by maximum projection images that combine the pixel intensities in all the z-planes of the cells (Fig. 1a). Since the hypoxia mimetic CoCl_2_ has also been shown to initiate a hypoxic response in cells^21^, we treated cells with CoCl_2_. Unlike hypoxia, we observed an increase in FN fibril containing cells upon CoCl_2_ treatment compared to the untreated control (Fig. 1a). Interestingly, in CoCl_2_ treated cells, images of individual z-planes showed more prominent regions of FN in the plane of the cell membrane, as detected from the cell membrane localization of β-catenin in Caki-1 cells^22^ (Fig. 1b). Percentage colocalization between β-catenin and FN at the cell membrane was also significantly increased (****P < 0.0001) in CoCl_2_ treated cells (Fig. 1b). Since the hypoxia mimetic CoCl_2_ and hypoxia (1% O_2_) differently affected the FN matrix organization, we wanted to confirm whether the increase in fibril assembly observed in CoCl_2_ treated cells was dose dependent. We treated cells with 1 µM, 10 µM and 50 µM CoCl_2_ for 18 h (Fig. 1c) and counted the number of fibril-containing cells by immunofluorescence staining of FN. We observed a significant increase (*P < 0.05, ***P < 0.001) in the number of fibril-containing cells at 10 µM and 50 µM CoCl_2_ respectively (Fig. 1d). Since fibril FN is characterized by its insolubility in deoxycholate^23^, we confirmed this data biochemically, by fractionating and quantifying the fibril fraction relative to the soluble fraction based on differential solubilities of the two fractions in deoxycholate. As shown in Fig. 1e the ratio of the fibril versus the soluble dimeric FN increased considerably in cells treated with 50 µM CoCl_2_. To evaluate if the increase in FN fibrils upon treatment with CoCl_2_, and the decrease in response to hypoxia, are due to changes in total FN protein levels, we measured total FN protein levels in response to hypoxia and CoCl_2_. In line with previously published data in normal renal epithelial cells^20^, FN deposition increased (*P < 0.05) in response to hypoxia but total FN levels in response to CoCl_2_ treatments remained unchanged (Fig. 1f,g). These data demonstrate that CoCl_2_ and hypoxia differently affect the FN matrix by respectively increasing fibril assembly in response to the CoCl_2_ whilst decreasing FN assembly under hypoxia.

### Cell migration in Caki-1 cells increases under hypoxia but remains unchanged by CoCl_2_ treatment

**Figure 2:**
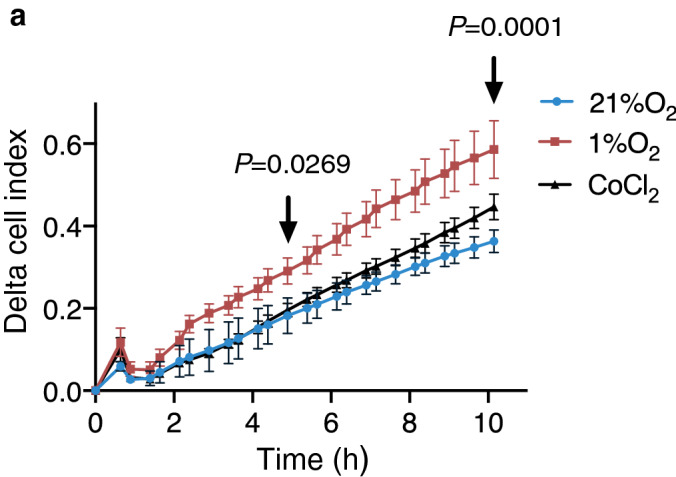
Hypoxia increases chemotactic migration. (**a**) Caki-1 cells were allowed to migrate in 21% O_2,_ hypoxia (1% O_2_) or in the presence of 50 µM CoCl_2_ towards a serum chemotactic gradient for 10 h. Real-time migration of the cells through the 8 µm pore was automatically quantified by the ACEA xCELLigence system and the migration curve was plotted. Each data point is an average of six technical replicates ± SD. Data is representative of three independent determinations. Statistically significant differences in migration between 21% O_2_ and 1% O_2_ at 5 h and 10 h (black arrows) are denoted by actual *P* values determined using two-way ANOVA (mixed-model).

To investigate the significance of the different responses of the matrix to hypoxia and CoCl_2_, we performed real-time cell migration assays between the two treatments. Migration of epithelial cells are guided primarily by the deposition and stiffness of the ECM laid down by fibroblasts and has been used as one of the indicators of metastatic propensity. However, we and others have previously shown that the FN matrix assembled by epithelial cells can in turn influence the migratory potential of the epithelial cells themselves^14,24^. Using a two-chamber set-up, we tracked the migration of Caki-1 cells exposed to 1% O_2_, 21% O_2_ or treated with CoCl_2_, towards a serum chemotactic gradient in real-time. Upon treatment with CoCl_2_, we observed no significant difference in migratory capacity compared to untreated cells (21% O_2_) over a period of 10 h. In contrast, migration under hypoxia was significantly increased compared to cell migration at 21% O_2_ as early as 5 h (*P < 0.05) and remained consistently increased at 10 h (**P < 0.01) (Fig. 2a).

### FN reorganization in response to CoCl_2_ or hypoxia is HIFα-independent

**Figure 3:**
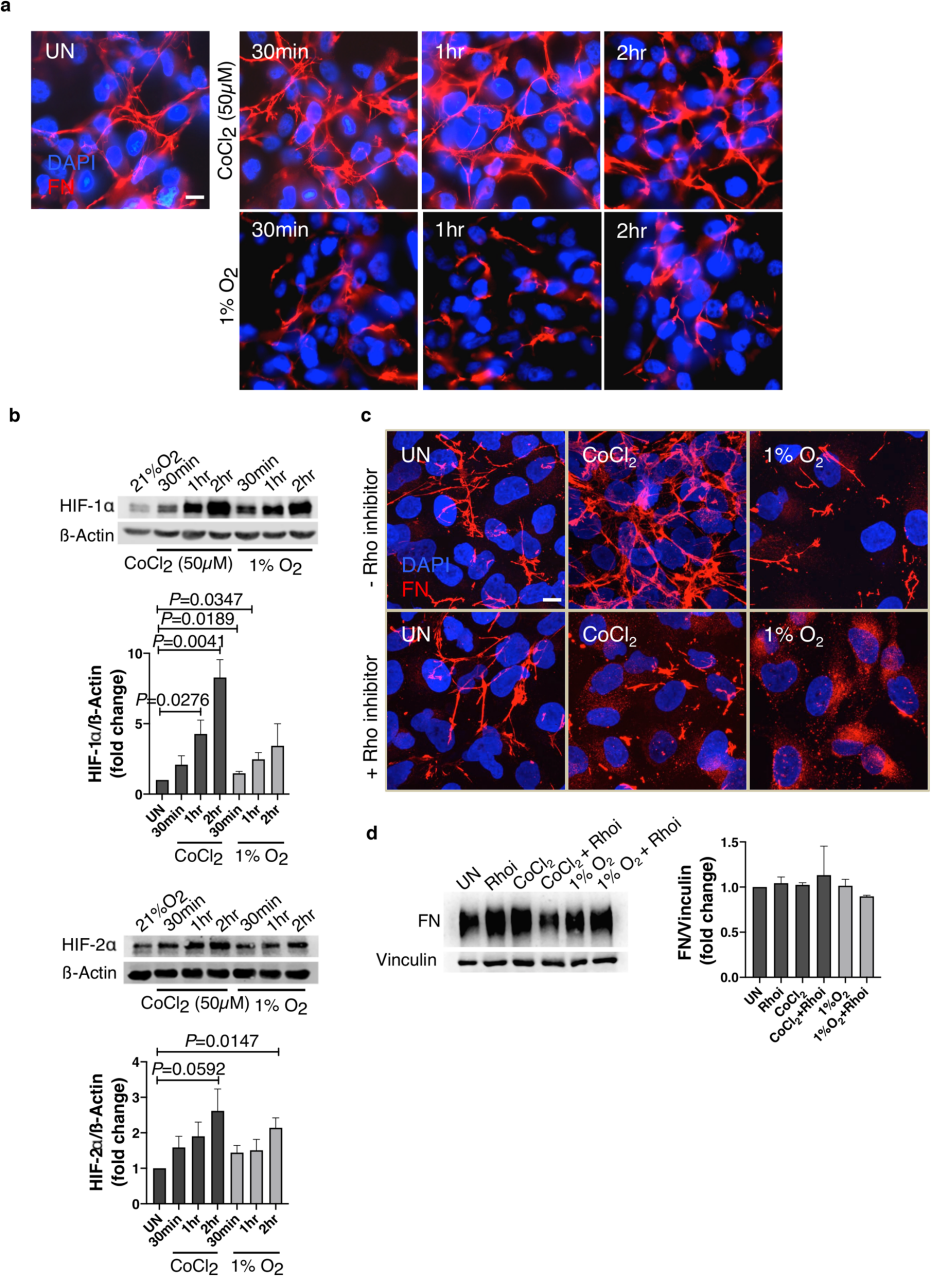

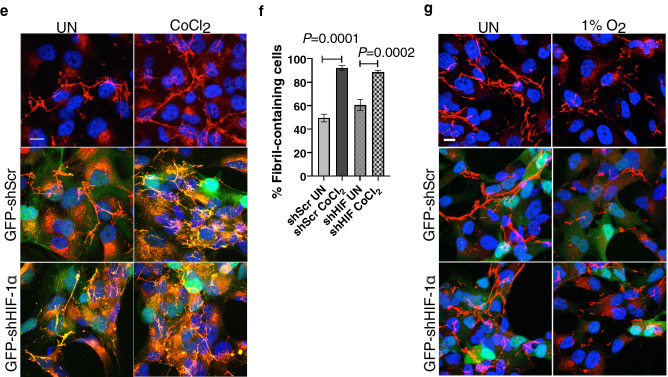
FN fibril assembly and disassembly in response to CoCl_2_ and hypoxia is HIFα independent and occurs within 30 min. (**a**) Caki-1 cells cultured at 21% O_2_ (UN), exposed to 1% O_2_ or treated with 50 µM CoCl_2_ for the indicated times were immunostained for FN (red) and counterstained with the nuclear stain DAPI (blue). (**b**) Cells treated as in (**a**) were lysed and immunoblotted for HIF-1α and HIF-2α. β-Actin was used as the loading control. Quantification of immunoblots represented as bar graphs are averages of three independent trials ± SEM. Statistical significance and actual *P* values were determined using the unpaired Student’s t-test. (**c**) Caki-1 cells pretreated with 1 µg/ml Rho inhibitor (+ Rho inhibitor) or without the Rho inhibitor (−  Rho inhibitor) were exposed to 1% O_2_ or treated with 50 µM CoCl_2_ for 2 h and immunostained for FN (red) and DAPI (blue). Scale bar = 10 µm. (**d**) Cells treated as in (**c**) were lysed and immunoblotted for total FN. Vinculin was used as the loading control. Quantification of FN levels normalized to vinculin is shown on the right. Bar graph is an average of three independent trials ± SEM. (**e**) Caki-1 cells transduced with shScr (200 MOI) or shHIF-1α (2000 MOI) expressing the GFP reporter were left untreated or treated with CoCl_2_ for 2 h and immunostained for FN (red) and DAPI (blue). Transduced cells are shown in green (GFP reporter). (**f**) Cells treated as in (**e**) were quantified for % of ‘fibril-containing’ cells and represented as bar graphs. Only GFP reporter-expressing virus transduced cells were included in the analysis. Quantification is an average of two independent experiments ± SEM. Statistical significance and actual *P* values were determined using the unpaired Student’s t-test. (**g**) Cells were transduced as in (**e**) and left untreated or exposed to 1% O_2_ for 2 h and immunostained for FN (red) and DAPI (blue). Scale bar = 10 µm.

Since treatments with CoCl_2_ and 1% O_2_ differently impacted migratory capacity, we next sought to investigate the mechanisms that contribute to fibril assembly in response to CoCl_2_ and disassembly in response to 1% O_2_. Assembly of FN into fibrils is a rapid process, shown to occur at 30 min by light microscopy and total internal reflection fluorescence (TIRF) techniques, at the resolution limits of conventional optical microscopy detection (~ 200 nm)^25,26^. Therefore, to investigate the kinetics of fibril assembly in response to CoCl_2_ or disassembly in response to hypoxia, we treated cells with CoCl_2_ or hypoxia, for 30 min, 1 h and 2 h. In CoCl_2_ treated cells, we found that fibrils assembled to reflect an increase, as early as 30 min. With similar kinetics, hypoxia exposure resulted in the disassembly of FN fibrils as early as 30 min (Fig. 3a). Since the cells responded swiftly to the hypoxia mimetic CoCl_2_ and to hypoxia, we next confirmed the hypoxia receipt in these cells by quantifying HIFα levels. We detected increased HIF-1α and HIF-2α protein levels (Fig. 3b) and upregulated HIFα transcript levels in cells exposed to either hypoxia or CoCl_2_ (Supplementary Fig. S1). Transcript levels of VEGFA, a HIF-1α transcriptional target^27^ also showed significantly increased levels 30 min post hypoxia or CoCl_2_ treatments (*P < 0.05) confirming HIF-1α activity in response to both treatments (Supplementary Fig. S1).

In RCC and other cell lines, small G protein RhoA activation has been shown to mediate FN fibril assembly by increasing cell contractility^12,28^. To test if Rho activation is required for CoCl_2_-mediated increase in FN fibrils, we quantified RhoA mRNA levels in response to CoCl_2_ and hypoxia. We observed a significant increase in RhoA mRNA levels (*P < 0.05) (Supplementary Fig. S2). We then treated cells with CoCl_2_ or hypoxia in the presence of the Rho inhibitor C3 transferase. Although preincubation with the Rho inhibitor for 2 h did not alter the fibril organization in cells, CoCl_2_ treatment in the presence of the Rho inhibitor abrogated the increase in FN fibril assembly, without changing total FN protein levels in these cells (Fig. 3c,d).

However, hypoxia in combination with the Rho inhibitor, revealed a noticeable disassembly of fibrils compared to hypoxia exposure alone (Fig. 3c). Since RhoA has been shown to require HIF-1α for activation in breast cancer cells and fibroblasts^29^, we investigated if HIF-1α is required for FN fibril assembly in response to CoCl_2_ treatment or the disassembly of fibrils in response to hypoxia. To test this, we knocked down HIF-1α using shHIF-1α expressing the GFP reporter, by adenovirus-mediated transduction. We obtained 70% HIF-1α knockdown as verified by immunoblotting (Supplementary Fig. S3, compare lanes 2 and 6). Knockdown in shHIF-1α transduced cells was additionally confirmed by detection of reduced HIF-1α fluorescence by immunostaining (Supplementary Fig. S4). We transduced cells with shScr or shHIF-1α for 24 h, followed by treatment for 2 h with CoCl_2_ or hypoxia. Despite HIF-1α knockdown, we observed a significant increase in the number of fibril-containing cells in response to CoCl_2_ treatment (***P < 0.001) (Fig. 3e,f). Similarly, HIF-1α knockdown also did not prevent the hypoxia-mediated decrease in FN fibrils (Fig. 3g). The absence of Akt pathway hyperactivation at high levels of virus infectivity (> 1000 MOI) (Supplementary Fig. S5) indicated that the cellular readout is due to depleted HIF levels and not an indirect effect of Akt activation.

To test whether HIF-2α may be involved in fibril assembly or disassembly in response to CoCl_2_ treatment or hypoxia, we knocked down HIF-2α using shHIF-2α expressing the GFP reporter, by adenovirus-mediated transduction. We obtained 80% knockdown as verified by immunoblotting (Supplementary Fig. S3B, compare lanes 2 and 6). As shown in Supplementary Fig. S6, HIF-2α knockdown did not prevent fibril assembly in response to CoCl_2_ treatment or the disassembly in response to hypoxia.

These results indicate that FN matrix assembly in response to CoCl_2_ or disassembly in response to hypoxia is Rho-dependent but independent of HIFα.

### HIF stabilization is insufficient to increase or decrease FN assembly

Although our data suggests that HIFα may not be involved in CoCl_2_ mediated FN fibril assembly or hypoxia mediated fibril disassembly (Fig. 3e–g, Supplementary Fig. S6), we wanted to confirm if HIFα stabilization, the functional read-out for hypoxia mimetics, is sufficient to reorganize fibril assembly. Hypoxia mimetics that are currently being used as an approach to establish experimental hypoxia, do so by inhibiting PHD2, the typical hydroxylase known to hydroxylate and target HIFα for proteasomal degradation^30,31^.

**Figure 4:**
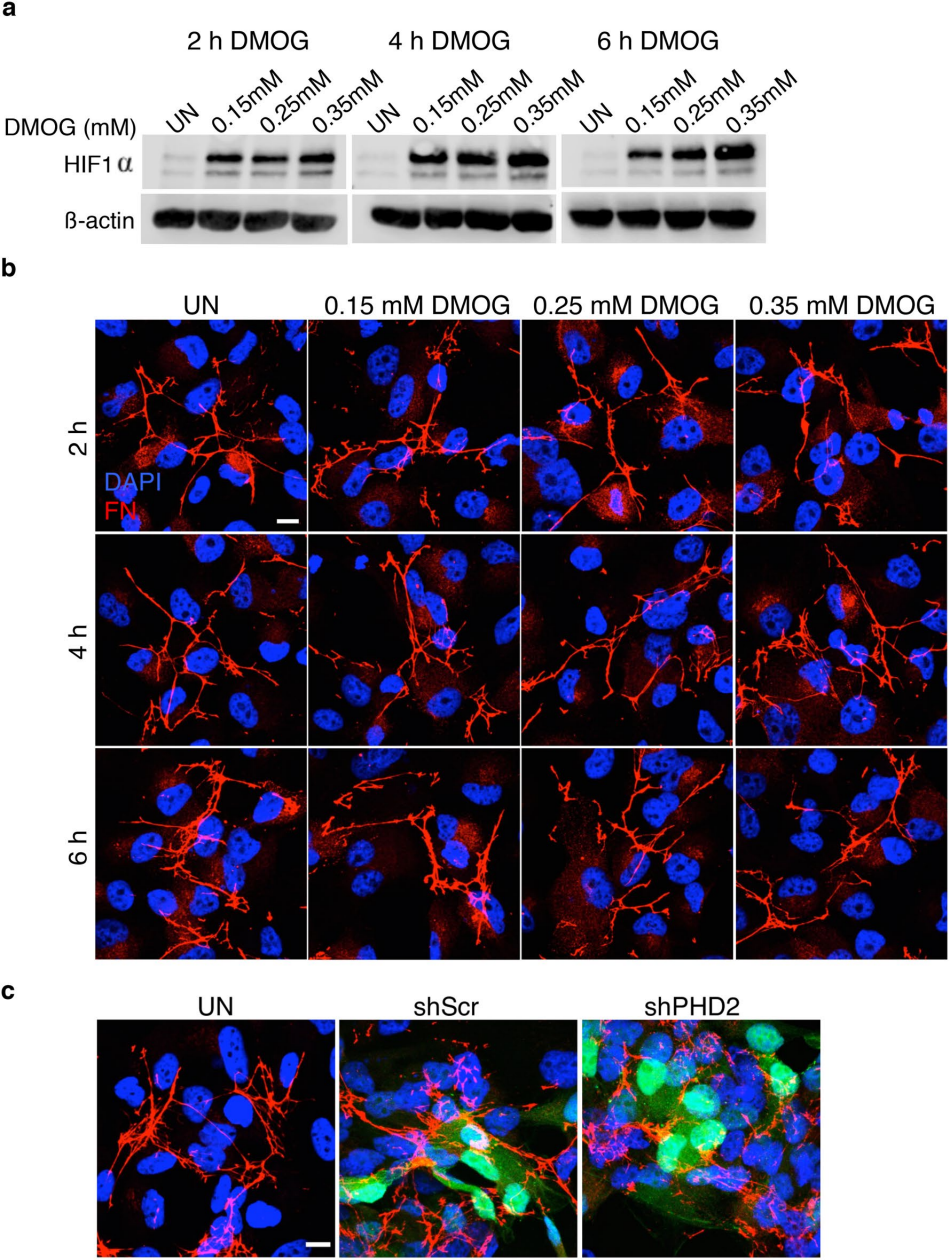
DMOG treatment or PHD2 knockdown does not affect fibril assembly. (**a**) Caki-1 cells treated with different concentrations of DMOG at the indicated times were lysed and immunoblotted for HIF-1α. β-Actin was used as the loading control. (**b**) Cells treated as in (**a**) were immunostained for FN (red) and counterstained with the nuclear stain DAPI (blue). Scale bar = 10 µm. (**c**) Caki-1 cells transduced with shScr (200 MOI) or shPHD2 (2000 MOI) expressing the GFP reporter were immunostained for FN (red) and DAPI (blue). Transduced cells are shown in green (GFP reporter).

HIFα PHDs (PHD1, PHD2 and PHD3) that hydroxylate HIFα, require Fe^2+^ and ascorbate as cofactors as well as 2-oxoglutarate and oxygen as co-substrates for their activity^32^. DMOG is a non-specific PHD inhibitor like CoCl_2_ but inhibits PHD activity by competing with the endogenous 2-oxoglutarate to stabilize HIFα^33,34^. Since CoCl_2_ treatment and 1% O_2_ stabilizes HIFα, we wanted to investigate whether HIFα stabilization by PHD inhibition using DMOG (Fig. 4a) would alter FN fibril assembly. We observed no significant difference in FN fibrillogenesis in DMOG treated cells compared to the untreated controls (Fig. 4b).

Next to conclusively establish if PHD2 inhibition is required for the increase or decrease in fibril assembly, we knocked down PHD2 using shRNAs expressing the GFP reporter (Supplementary Fig. S7). We observed no significant differences in fibrillogenesis between the shScr and PHD2 knocked down cells (Fig. 4c) confirming that PHD2 inhibition has no independent effect on fibril assembly or disassembly in Caki-1 cells. These data indicate that the two different hypoxia mimetics that we examined, exert distinct effects on the FN matrix which are dissimilar to the FN fibril disassembly observed under 1% O_2_. These results reveal that although hypoxia mimetics and hypoxia inhibit PHDs and stabilize HIFα, 1% O_2_ specifically causes FN matrix disassembly by a mechanism that does not recapitulate the effects of hypoxia mimetics CoCl_2_ or DMOG.

### FN fibril assembly in response to CoCl_2_ requires integrins α4 and α5

**Figure 5:**
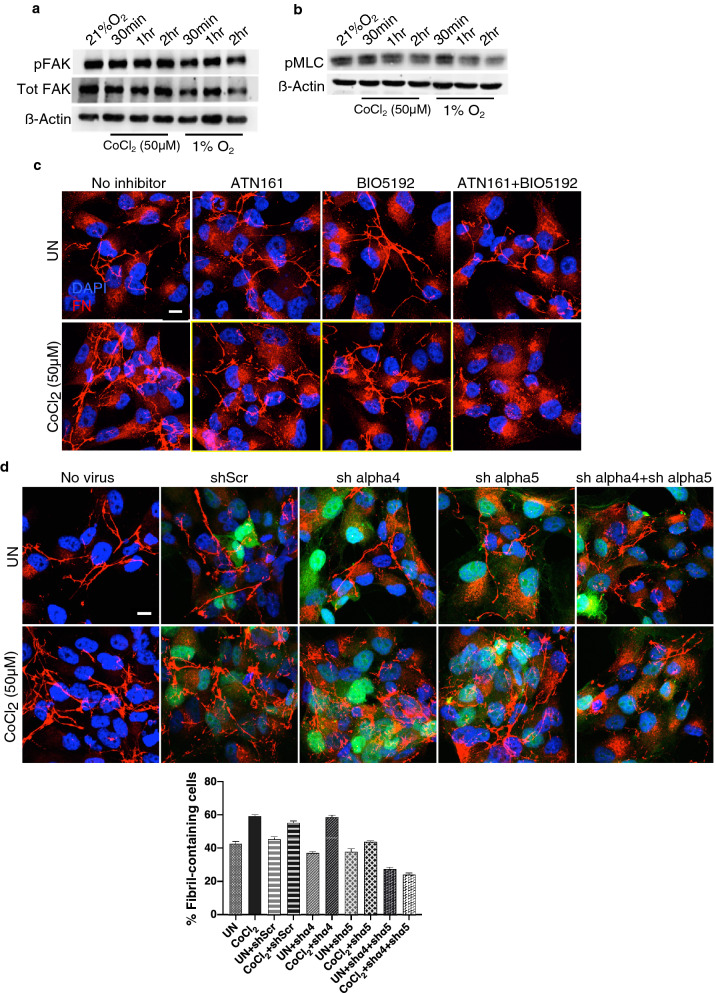
Integrins α4 and α5 are required for FN fibril assembly in response to CoCl_2_. (**a**) Caki-1 cells cultured at 21% O_2_, exposed to 1% O_2_ or treated with 50 µM CoCl_2_ for the indicated times. Cells were lysed and immunoblotted for pFAK and total FAK. β-Actin was used as the loading control. (**b**) Cell lysates as in (**a**) were immunoblotted for pMLC. β-Actin was used as the loading control. (**c**) Caki-1 cells were pretreated with 10 µM ATN-161 (Integrin α5 inhibitor), 1 µg/ml BIO5192 (Integrin α4 inhibitor) or a combination of both for 30 min followed by treatment with 50 µM CoCl_2_ for 2 h and immunostained for FN (red) and counterstained with the nuclear stain DAPI (blue). Scale bar = 10 µm. (**d**) Caki-1 cells transduced with shScr (200 MOI) or shalpha4 (2000 MOI), shalpha5 (2000 MOI) or a combination of shalpha4 (1000 MOI) and shalpha5 (1000 MOI) expressing the GFP reporter were left untreated or treated with 50 µM CoCl_2_ for 2 h and immunostained for FN (red) and DAPI (blue). Transduced cells are shown in green (GFP reporter). The bar graph below the figure quantifies the % ‘fibril-containing’ cells in GFP reporter-expressing virus transduced cells. Data is representative of two independent experiments. More than 80 cells were counted in each condition.

Using a combination of HIFα knockdown and stabilization approaches, our data indicated that fibril reorganization is independent of HIF-1α and HIF-2α (Fig. 3e–g, Supplementary Fig. S6). However, since RhoA inhibition abrogated FN fibril assembly in response to CoCl_2_ (Fig. 3c), we wanted to evaluate if mediators of the Rho pathway maybe involved in fibril assembly. One of the proteins involved in activation of RhoA-ROCK is focal adhesion kinase (FAK), which has been shown to undergo phosphorylation at Y397 in response to hypoxia in fibroblasts^29^. To test if CoCl_2_ treatment increased FAK phosphorylation, we treated cells with CoCl_2_ or exposed cells to hypoxia and quantified FAK phosphorylation. Neither treatments increased FAK phosphorylation in the cells (Fig. 5a).

Since Rho signal transduction has been shown to lead to myosin light chain (MLC) phosphorylation, we determined levels of phosphorylated myosin light chain (pMLC) in response to CoCl_2,_ treatment or hypoxia. Although phosphorylation of myosin light chain has been associated with fibril assembly^20^, we observed no quantifiable changes in the phosphorylation of myosin light chain kinase in CoCl_2_ treated cells. Hypoxia exposure also did not alter accumulation of pMLC in the cells (Fig. 5b). Furthermore, neither treatment reorganized actin filaments into stress fibers (Supplementary Fig. S8). These data collectively confirmed that mediators of the Rho pathway and stress fiber activation may not directly contribute to FN fibril assembly initiated by CoCl_2_ treatments.

The engagement of FN with integrin α5β1, converts the relaxed ligand–receptor binding to a tensioned state^35,36^ leading to cytoskeletal tension and stress fiber formation. Since our data did not reveal activation of the classical downstream events following integrin α5β1 engagement such as phosphorylation of MLC, FAK and stress fiber formation (Fig. 5a,b, Supplementary Fig. S8), we predicted that fibril assembly may instead involve engagement of integrin α4 which has been shown to increase fibril assembly without activation of stress fibers^37,38^. To test this, we treated cells with CoCl_2_ in the presence of BIO5192 an integrin α4 inhibitor or ATN161 an integrin α5 inhibitor or a combination of both. While we observed a slight reduction in the fibril response when treated with the inhibitors separately, we observed a noticeable decrease in FN fibril assembly only when the two inhibitors were combined (Fig. 5c). To confirm this data, we knocked down integrin α4 and integrin α5 using shRNAs expressing the GFP reporter by adenovirus-mediated transduction. Since we obtained 40% knockdown of integrin α4 by immunoblotting (Supplementary Fig. S9, compare lanes 1 and 6), we additionally verified knockdown by detection of reduced integrin α4 levels by immunostaining (Supplementary Fig. S9). Cells knocked down for integrins α4 and α5 combined, failed to increase FN fibril formation in response to treatment with CoCl_2_ (Fig. 5d). Percentage of fibril-containing cells considerably decreased when integrins α4 and α5 were knocked down in CoCl_2_ treated cells. Thus FN fibril assembly in response to CoCl_2_ requires the activation of integrins α4 and α5 receptors.

## Discussion

In this study, we investigate the regulation and significance of FN matrix assembly in response to hypoxia (1% O_2_) and the hypoxia mimetic CoCl_2_. Our results reveal that hypoxia significantly increases cell migration of metastatic renal cancer Caki-1 cells, fragments the epithelial FN matrix and increases FN deposition (Figs. 1,2). Although hypoxia and hypoxia mimetics primarily impact the activity of PHDs resulting in HIFα stabilization, we find that the hypoxia mimetic CoCl_2_ shows contrasting results to hypoxia on its effects on FN matrix organization (Fig. 1a). Not surprisingly, PHD2 inhibition by treatment with a commonly used hypoxia mimetic DMOG or knockdown of PHD2, despite HIFα stabilization, was unable to affect FN matrix organization (Fig. 4). Moreover, HIFα knockdown did not affect CoCl_2_-mediated fibril assembly or the hypoxia-mediated fibril disassembly (Fig. 3e–g, Supplementary Fig. S6). These findings collectively establish that FN reorganization by hypoxia and hypoxia mimetics is HIFα independent.

Hypoxia in the tumor microenvironment contributes to unfavourable disease outcome^39^ and HIF-1α target genes in RCC have been shown to increase migration and invasion of the primary tumor cells^40,41^. Our data indicates that stabilization of HIF-1α alone maybe insufficient to drive migration but instead requires the synergistic contribution of other signaling events driven by hypoxia in mediating this phenotypic transition. This is evident from our observation demonstrating that hypoxia increased chemotactic migration of renal cancer cells but the stabilization of HIF-1α in response to CoCl_2_ did not (Fig. 2).

Here we explore the mechanistic events that lead to fibril assembly and disassembly. We show that matrix formation in response to CoCl_2_ requires Rho activity and the participation of integrins α4 and α5 (Figs. 3c, 5c,d). The absence of pFAK and pMLC activation or the absence of stress fibers (Fig. 5a,b, Supplementary Fig. S8) concomitant with fibril formation, indicate that downstream events associated with cell tension are not required to mediate this process. Interestingly, in response to hypoxia, Rho inhibition resulted in greater fragmentation of the fibril matrix, compared to hypoxia alone. In these cells and in cells treated with CoCl_2_ and Rho inhibitor combined, FN appeared to accumulate in close proximity to the nucleus (Fig. 3c). Since total FN levels remain comparable between Rho inhibitor treated versus untreated cells (Fig. 3d), we predict that Rho may be involved in the trafficking of FN in response to hypoxia and CoCl_2_ treatments. We have previously shown that FN is internalized and recycled to the cell surface in response to TGF-β^24^. Since TGF-β treatment in normal kidney cells increases cell tension and stress fiber formation^20^, it is likely that changes in cell tension as seen in our experiments with Rho inhibition, affects the process of trafficking that is necessary for the assembly or disassembly of FN in response to CoCl_2_ or hypoxia treatments. To explore this possibility of intracellular trafficking, we coimmunostained FN with the golgi marker GM130 (Supplementary Fig. S10). FN localization to the golgi is known and altered colocalization of the two proteins would indicate the dynamic regulation of FN in response to hypoxia and CoCl_2_ when cell tension is inhibited. The significant increase in FN-GM130 colocalization in hypoxia and CoCl_2_ treated cells upon Rho inhibition (Supplementary Fig. S10) indicated that intracellular trafficking controlled by cell tension may play a critical role in driving fibril formation and disassembly. Although these findings are preliminary, the results nevertheless raise the interesting possibility of the role of cell tension in determining the localization of FN during hypoxic states.

While we were unable to point to a specific mechanistic cascade leading upto the disassembly of the FN matrix during hypoxia, our data suggests that this process is HIFα independent and not accompanied by significant changes in stress fiber activation and FAK or MLC phosphorylation. It is very likely that suppressed activation of integrins α4 and α5 maybe involved. The lack of available antibodies that detect active conformations of integrin α4 prevented us from testing this possibility.

The transition of epithelial cells to metastatic phenotypes are regulated by FN matrix assembly^24,42^ and autocrine production of FN by the tumor cells^43^. Addition of FN to 3D cultures of normal breast cells encourages lumen filling in the acini culture model mimicking early stage epithelial tumors^44^. The administration of a 76aa peptide derived from the first Type III repeat of FN that accelerates FN fibril formation has been shown to specifically reduce metastasis in xenograft mouse models of breast cancer^14^. We have also previously demonstrated that the assembly state of FN determines migratory potential of normal breast cells^24,42^. Thus the reorganization of FN in epithelial cells plays a critical role in influencing cellular phenotypes in multiple experimental models of cancer.

Considering the functional significance of FN matrix assembly and disassembly in driving disease outcome, our study has examined the factors that contribute to matrix reorganization and evaluated for the first time the disparate roles of hypoxia mimetics from tumor hypoxia in impacting cancer progression.

## Materials and methods

### Cell lines and culture conditions

Metastatic Caki-1 renal cancer epithelial cells were obtained from the American Type Culture Collection (ATCC, #HTB-46) and cultured in McCoy5A media (ATCC, #30-2007) supplemented with 10% FBS (Hyclone, #SH30109.03) and 1% Penicillin–Streptomycin (Gibco, #15140-122). Cells were maintained in a 37 °C humidified incubator buffered with 5% CO_2_. All experiments were performed at 70–80% cell densities, which corresponded to 2.6 × 10^6^ cells/75 cm^2^ culture area.

### Antibodies and reagents

Antibodies for immunoblotting and immunocytochemistry: GAPDH (Invitrogen, #437000), HIF-1α (Cell Signaling Technology, #14179S), HIF-2α (Abcam, #ab207607), Fibronectin (IB) (Abcam, #ab23750), Fibronectin (ICC) (SCBT, #sc-59826), β-Actin (Invitrogen, #MA1-140), Phospho-FAK (Life Technologies, #396500), Phospho-MYL9 (Invitrogen, #PA5-17727), Total-FAK (Invitrogen, #39-6500), Vinculin (Abcam, #129002), Integrin α4 (Abcam, #ab202969), Integrin α5 (Abcam, #ab150361), GM130 (BD, #610823), Alexa Fluor 568 (Invitrogen #A10037).

Cobalt chloride hexahydrate (MP Biomedicals LLC, #194642) stock solutions were made fresh on the day of the experiment and used at working concentrations of 1 µM, 10 µM and 50 µM. Rho inhibitor (Cytoskeleton, #CT04) was used at a working concentration of 1 µg/ml. Integrin α5 inhibitor ATN-161 (MedKoo Biosciences, #200350a) and Integrin α4 inhibitor BIO5192 (Tocris, #5051) were reconstituted and stored according to manufacturer’s recommendations. DMOG (Abcam, #ab141586) was used at a working concentration of 0.15 mM, 0.25 mM and 0.35 mM for 2 h, 4 h and 6 h.

### Adenovirus mediated knockdown

Pre-validated Ad-GFP-U6-h-HIF-1α-shRNA, Ad-GFP-U6-scrmb-shRNA, Ad-GFP-U6-h-EGLN1-shRNA, Ad-GFP-U6-h-ITGA4-shRNA and Ad-GFP-U6-h-ITGA5-shRNA were purchased from Vector Biosystems Inc. at a viral titer of 1.9 × 10^10^ PFU/ml. We obtained ~ 80% knockdown at 2000 MOI.

shHIF-1α sequence:

5′-CCGG-GTGATGAAAGAATTACCGAATCTCGAGATTCGGTAATTCTTTCATCAC-TTTTT-3′

shScr sequence:

5′-CAAC-AAGATGAAGAGCACCAA-CTCGAG-TTGGTGCTCTTCATCTTGTTG-TTTTT-3′

shEGLN1 sequence:

5′-CCGGGACGACCTGATACGCCACTGT-CTCGAG-ACAGTGGCGTATCAGGTCGTC-TTTTT 3′

shPHD2 sequence:

5′-CCGGGACGACCTGATACGCCACTGT-CTCGAG-ACAGTGGCGTATCAGGTCGTC-TTTTT-3′

shITGA4 sequence:

5′-CCGGCGGGAGCAGTAATGAATGCAA-CTCGAG-TTGCATTCATTACTGCTCCCG-TTTTT-3′

shITGA5 sequence:

5′-CCGGCTCCTATATGTGACCAGAGTT-CTCGAG-AACTCTGGTCACATATAGGAG-TTTTT-3′

### RNA extraction and qPCR

Total RNA was extracted from 500,000 cells using the Qiagen RNeasy kit (#74104) according to manufacturer’s protocol. RNA quality was assessed using the Nanodrop and 550 ng RNA was used to create cDNA using the TaqMan Reverse Transcription kit (N808-0234). 10 ng cDNA was used in duplicate qPCR reactions using master mix from TaqMan Universal PCR Master Mix (#4324018) and TaqMan primer probes. The following primer probes that spans exons were used to quantify mRNA levels: RHOA #HS00236938_M1, HIF-1α, #HS00153153_M1, HIF-2α #HS01026149_M1. qPCR was performed on an Applied Biosystems 7500 SDS real time PCR system. GAPDH primer probes were used for normalization. Relative expression was calculated using the delta Ct method.

### DOC assay

Deoxycholate (DOC) fractionation was carried out as previously described^24,42^. Briefly, cells were washed in ice-cold phosphate-buffered saline (PBS) and soluble FN protein extracted in buffer containing 2% deoxycholate and 0.02 M Tris–HCl, pH 8.8, supplemented with 2 mM phenylmethylsulphonyl fluoride, 2 mM EDTA, 2 mM iodoacetic acid and 2 mM N-ethylmalemide. Following lysis for 30 min at 4 °C, the lysates were centrifuged at 15,000 rpm for 30 min to separate the soluble FN in the supernatant. The pellet fraction containing the fibril FN was resuspended in SDS lysis buffer containing 1% SDS, 25 mM Tris–HCl, pH 8.0 and protease inhibitors. The pellet fraction containing the SDS lysis buffer was heated for 1 min at 95 °C. The entire fibril fraction was loaded on a 5% polyacrylamide gel and 10% of the soluble fraction was loaded alongside. The soluble FN and pellet FN were normalized to GAPDH. Average fold differences in the ratio between normalized fibril and non-fibril fractions were plotted as bar graphs.

### Protein extraction and Immunoblotting

Protein extraction was performed at 4 °C using ice-cold SDS lysis buffer containing protease and phosphatase inhibitors (1 mM DTT, 1 mM EDTA, 100 µg/ml PMSF, 1 µg/ml Leupeptin, 1 mM Sodium orthovanadate). Proteins were separated by SDS-Poly acrylamide gel electrophoresis and immunoblotted for specific proteins as indicated. Vinculin, β-Actin, β-Tubulin and GAPDH were used as loading controls.

Quantification of immunoblots was performed using the Li-Cor Image Studio Software version 5.2. Pixel intensities of each protein normalized to the loading control were averaged from multiple independent experiments and fold-differences between untreated and treated samples were plotted as shown. *P* values were determined using the Student’s t-test.

### Immunocytochemistry and imaging

For immunocytochemistry of FN, cells were seeded on sterile coverslips in a 6-well plate at a density of 173,000 cells per well. The coverslips were fixed in 4% paraformaldehyde and permeabilized in 0.1% Triton X-100 on ice for 1 min. After blocking in 5% BSA-1 × PBS, FN antibody (1:70) was added for 1 h followed by 1 h incubation with secondary antibody. After repeated washing in 1 × PBS, cells were stained with the DNA stain DAPI (4,6-diamidino-2-phenylindole dihydrochloride) (Roche #1023627001) and mounted using Prolong gold anti-fade mount media (Invitrogen #P36930) on glass slides. Imaging and z-stacks (1 µm z-slice) were acquired using a Leica TCS SPEII confocal microscope at consistent acquisition parameters for each experiment.

### Fibril count

Plots in Figs. 1d, 3f, 5d and Supplementary Fig. S6B were constructed as previously described where a FN track ~ 3 µm in length is considered a fibril^24^. Cells containing such threshold lengths of FN were counted as “with fibril”. Random fields of view within each sample were included in our analysis to quantify the percentage of fibril-containing cells. A total of at least 100 cells were counted in each condition. Data in figures that include fibril counts are representative of 3 independent experiments. Paired Student’s t-test was used to calculate *P* value.

### Phalloidin staining

For stress fiber detection using Phalloidin, the cells were rinsed in 1 × ice-cold PBS and fixed in 4% formaldehyde for 15 min. After subsequent washes in 1 × PBS, Phalloidin (Cell Signaling Technology #13054S) was added to the cells at a dilution of 1:200 according to manufacturer’s instructions. Phalloidin was incubated for 15 min at room temperature. After incubation, cells were washed with 1 × PBS and mounted on glass slides using the Prolong gold anti-fade mount media. The slides were imaged using confocal microscopy.

### Cell migration

Real-time cell migration was performed in 16-well CIM plates using the ACEA xCELLigence instrument. To set-up the cell migration assay, 160 µl of serum-containing media was plated in the lower chamber of the CIM plates and topped with 50 µl serum-free media to equilibrate the 8 µm pore sized membrane that separates the top and bottom chambers. For a negative control, one triplicate set of wells contained serum-free media in the lower chamber. After equilibration of the membrane for 1 h, 30,000 cells in 100 µl serum-free media were plated on the top chamber of the CIM plate. After the plate is assembled in the instrument, cell migration from the upper to lower chambers is quantified by measurement of cell impedance. Impedance is created when cells travel through the membrane lined with gold electrodes. As cells migrate, they impede the flow of current between the electrodes to generate impedance values, which are converted by the xCELLigence software to cell index (cell numbers). For cell migration at 1% O_2_, the xCELLigence equipment was placed in a hypoxia chamber and cell migration was performed.

Cell index values generated by the instrument were analyzed using the RTCA DP software Pro. The values were exported to Excel and migration curves plotted. Each treatment condition was performed in six technical replicates and each experiment was performed three times.

### Rho inhibitor assay

For Rho inhibition, cells were pre-treated with the Rho inhibitor at a concentration of 1 µg/ml for 2 h. Pre-treatment was followed by treatment with CoCl_2_ or exposure to 1% O_2_ for 2 h before the cells were fixed and processed for immunocytochemistry or directly lysed in SDS lysis buffer for immunoblotting.

### Integrin inhibition

Integrin α5 and integrin α4 inhibitor experiments were performed by pre-treating cells with 10 µM ATN-161 (Integrin α5 inhibitor), 1 µg/ml BIO5192 (Integrin α4 inhibitor) or a combination of both for 30 min. Pre-treatment was followed by treatment with 50 µM CoCl_2_ for 30 min in the presence of the inhibitor. The cells were fixed and stained for FN and analyzed by confocal microscopy.

### Statistical analysis

Cell migration assays and qPCR assays were run in technical and biological replicates. All other assays were performed as multiple independent trials and represented as mean ± SEM. Statistical significance between untreated and treated samples for FN fibril counts, immunoblot intensities and qPCR analyses were calculated using the unpaired Student’s t-test. Statistical analyses for migration kinetics were calculated using two-way ANOVA (mixed model). Significance (*P* value) was determined using GraphPad Prism 8.

## Supplementary information

Supplementary Figures

## Data Availability

All data generated or analyzed during this study are included in this published article (and its Supplementary information files).
