# Supplementary material for: HIFα independent mechanisms in renal carcinoma cells modulate divergent outcomes in fibronectin assembly mediated by hypoxia and CoCl2
Source: Sci Rep. 2020 Oct 29;10:18560. doi: 10.1038/s41598-020-75756-5 (PMC7596723; doi:10.1038/s41598-020-75756-5)

**Title**: **HIFα independent mechanisms in renal carcinoma cells modulate divergent outcomes in fibronectin assembly mediated by hypoxia and $CoCl_2$**

**Authors:** Carina Magdaleno[1], Leah Dixon[1], Narendiran Rajasekaran[1] and Archana Varadaraj[1*]

**Affiliations:** Department of Chemistry and Biochemistry, Northern Arizona University, Flagstaff 86004 Arizona, USA[1]

**Correspondence to*:** Archana Varadaraj, Department of Chemistry and Biochemistry, PO Box 5698, Building 36, Room 430, Northern Arizona University, Flagstaff 86004, Arizona, USA.
Email: archana.varadaraj@nau.edu

## Supplementary Figure S1

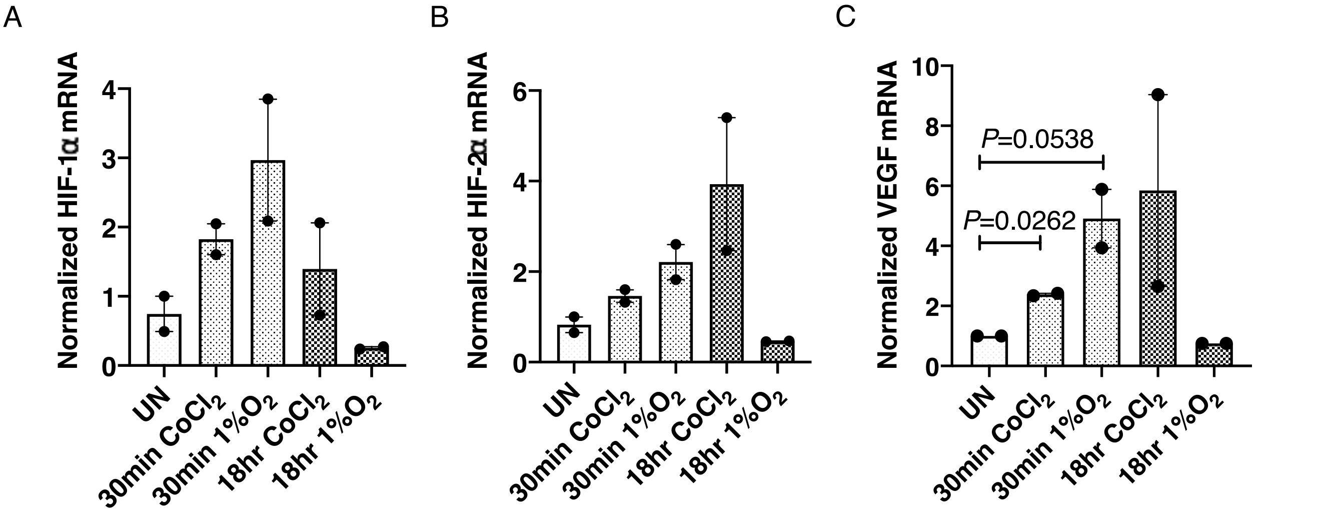


**Fig. S1**: **mRNA expression levels of HIFα and VEGF in cells untreated or treated with $CoCl_2$ and 1% $O_2$.**
Caki-1 cells were treated with 50$\mu$M $CoCl_2$ or exposed to 1% $O_2$ for the indicated times and cells processed for RNA extraction. Relative expression of HIFα (A,B) and VEGFA (C) was determined using the delta delta Ct method. GAPDH was used for normalization. Data is an average of two independent trials each performed in triplicates.

## Supplementary Figure S2

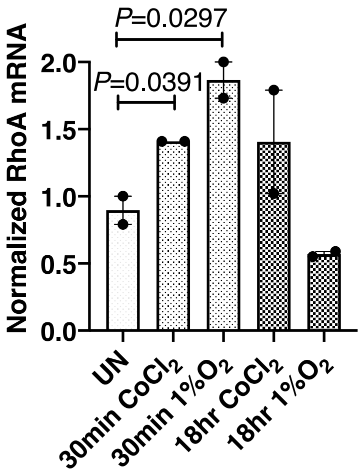


**Fig. S2**: **mRNA expression levels of RhoA in cells untreated or treated with $CoCl_2$ and 1% $O_2$.**
Caki-1 cells were treated with 50$\mu$M $CoCl_2$ or exposed to 1% $O_2$ for the indicated times and cells processed for RNA extraction. Relative expression of RhoA was determined using the delta delta Ct method. GAPDH was used for normalization. Data is an average of two independent trials each performed in triplicates. Statistical significance was determined using the unpaired t-test.

## Supplementary Figure S3

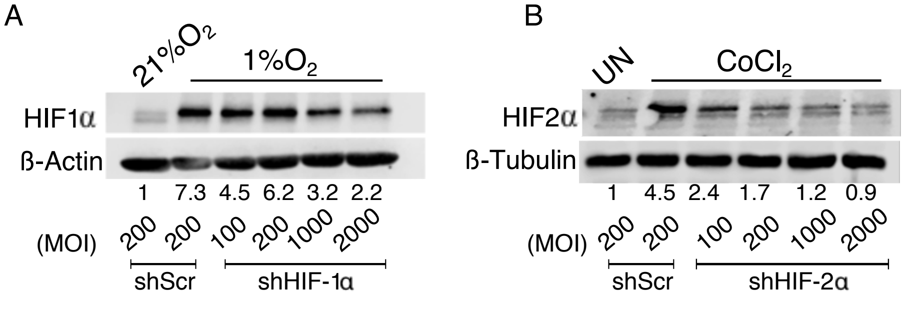


**Fig. S3**: **HIFα knockdown using adenovirus-mediated transduction.**
Caki-1 cells exposed to hypoxia (A) or 50$\mu$M $CoCl_2$ (B) were preincubated with Ad-GFP-HIFα shRNA for 24 h at different MOI as shown in the figure. At 2000 MOI we observed atleast 70% knockdown of the respective proteins. Lysates were probed for HIFα proteins and ß-actin or tubulin was used as respective loading controls.

**Supplementary Figure S4**

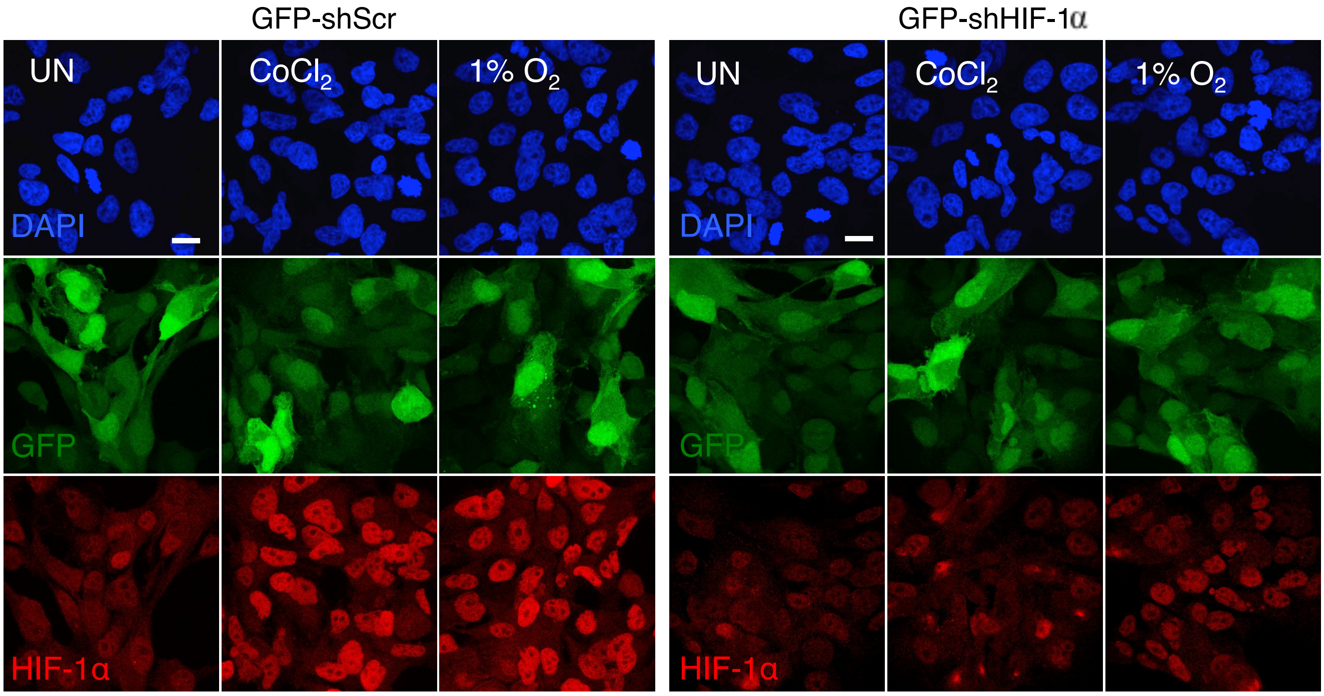


**Fig. S4 :HIF-1α knockdown in shHIF-1α transduced cells.**
Caki-1 cells were transduced with shScr (200 MOI) or shHIF-1α (2000 MOI) for 24 h and treated with $CoCl_2$ or exposed to 1% $O_2$ for 2 h as in Fig. S3. Cells were fixed and immunostained for nuclear stain DAPI (blue) and HIF-1α(red). Transduced cells are seen in green. Scale bar= 10$\mu$m. Images are confocal maximum projections acquired at identical image acquisition parameters in the different experimental conditions.

**Supplementary Figure S5**

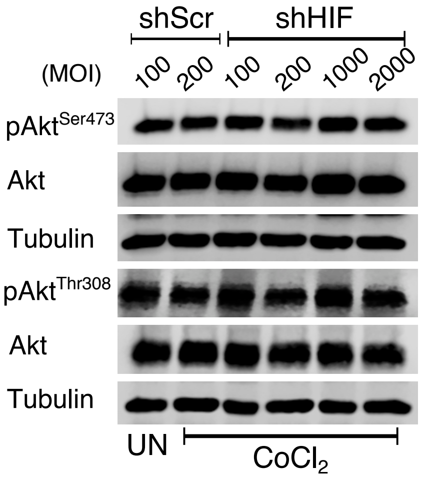


**Fig. S5 :Akt activation in shHIF-1α transduced cells.**
Caki-1 cells were transduced with shScr or shHIF-1α for 24 h and treated with $CoCl_2$ for 2 h as in Fig. S3. Transduced cells were lysed and immunoblotted for the proteins as shown.
.

## Supplementary Figure S6

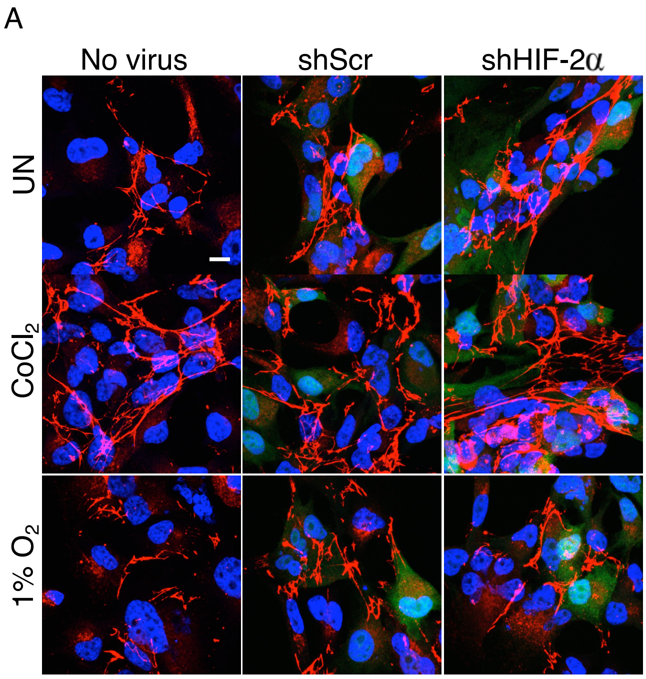


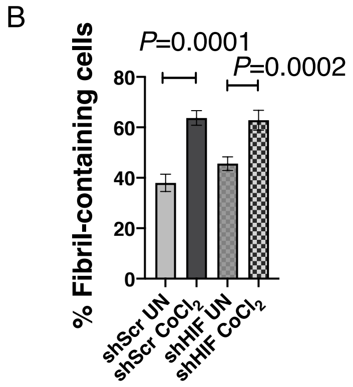


**Fig. S6**: **FN immunostaining in cells knocked down for HIF-2α**.

Caki-1 cells were transduced with shScr or shHIF-2α for 24 h and treated with $CoCl_2$ or 1% $O_2$ for 2 h. Transduced cells expressing the GFP reporter are shown in green. Cells were stained for FN (red) and counterstained with the nuclear stain DAPI (blue). (B) % FN fibril containing cells were determined in the transduced cells (GFP reporter) by counting using a confocal microscope. Data is an average of ~200 cells per condition. Statistical significance was determined using the unpaired t-test. Scale bar=10$\mu$m.

## Supplementary Figure S7

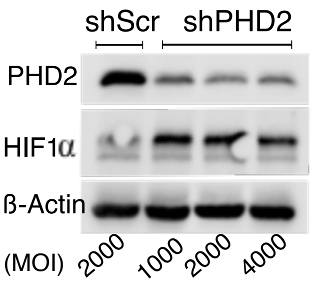


**Fig. S7**: **PHD2 knockdown using adenovirus-mediated transduction**.

Caki-1 cells were transduced with Ad-GFP-PHD2 shRNA for 24 h at different MOI as indicated. Lysates were probed for PHD2. Actin was used as the loading control.

## Supplementary Figure S8

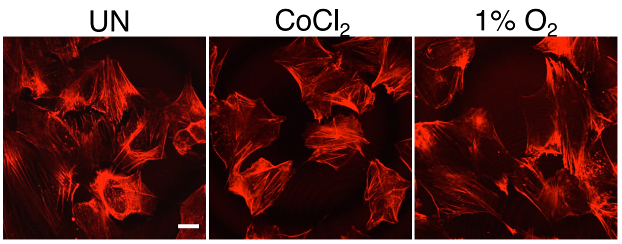


**Fig. S8**: **Phalloidin staining in cells untreated or treated with $CoCl_2$ and 1% $O_2$**.

Caki-1 cells treated with 50 $\mu$M $CoCl_2$ or exposed to 1% $O_2$ for 2 h were stained with Phalloidin to detect stress fibers. Scale bar=10$\mu$m.

## Supplementary Figure S9

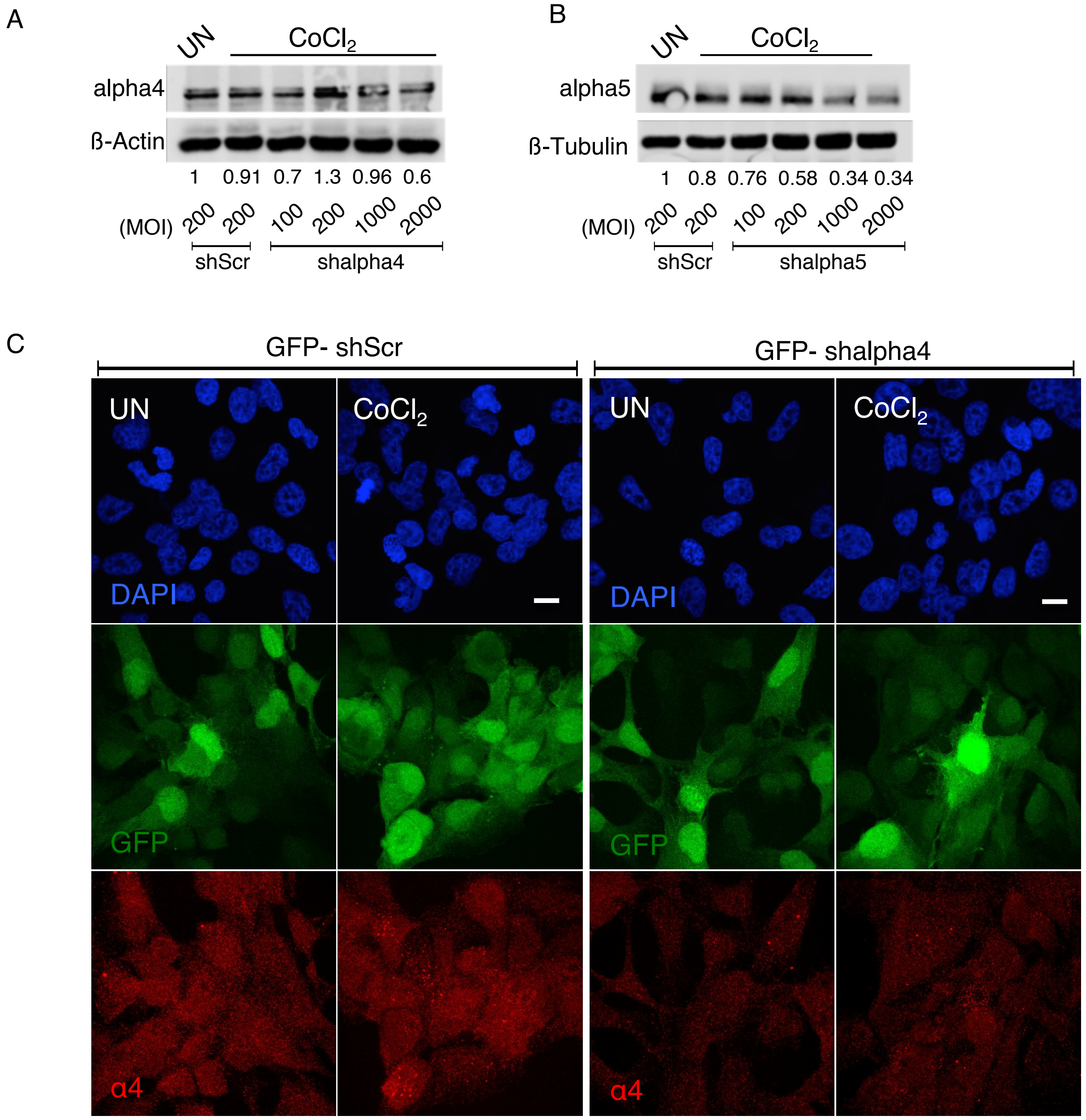


**Fig. S9**: **Integrin knockdown using adenovirus-mediated transduction**.
Caki-1 cells were transduced with Ad-GFP-alpha4 shRNA (A) or Ad-GFP-alpha5 shRNA (B) for 24 h at different MOI as indicated. Lysates were probed for integrin alpha4 and integrin alpha5 as shown. Actin and Tubulin were used as loading controls respectively. (C) Cells transduced with shScr (200MOI) or shalpha4 (2000 MOI) virus for 24 h were untreated or treated with $CoCl_2$ for 2 h and immunostained for integrin alpha4 (red) and counterstained with the nuclear stain DAPI (blue). Virus transduced cells expressing the GFP reporter are shown in green. Scale bar=10$\mu$m. Images are confocal maximum projections acquired at identical image acquision parameters in the different experimental conditions.

## Supplementary Figure S10

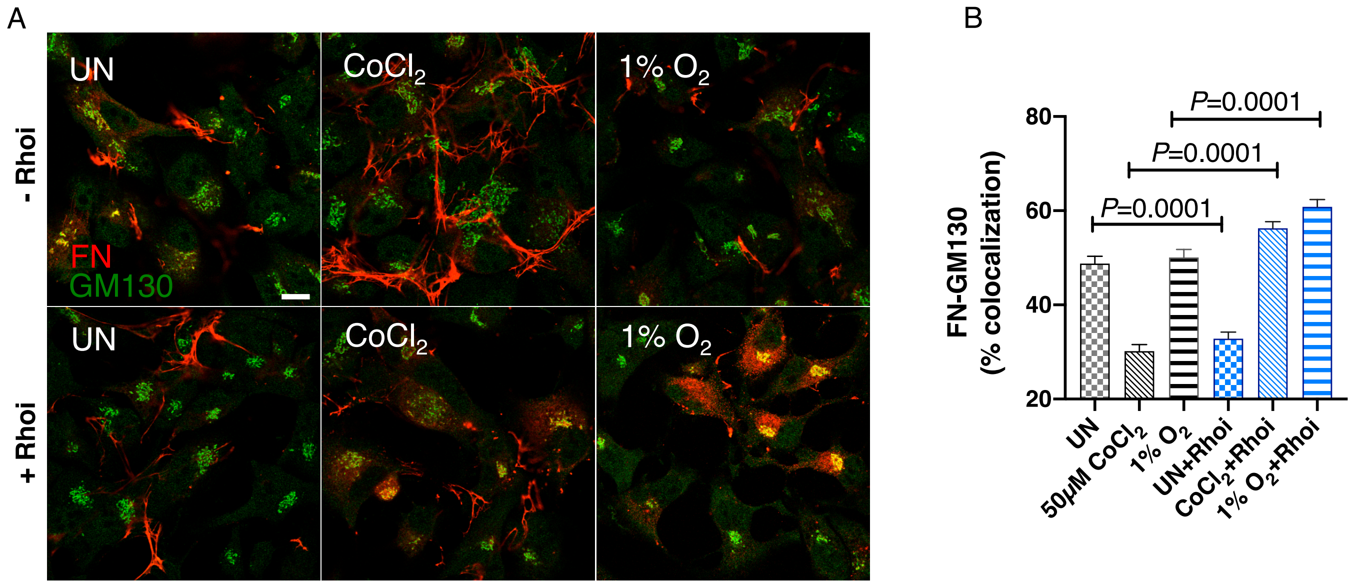


**Fig. S10**: **Localization of FN and the golgi marker GM130**.
(A) Caki-1 cells were treated with $CoCl_2$ or 1% $O_2$ for 2 h with or without the Rho inhibitor C3 transferase. Cells were immunostained for the golgi marker GM130 (green) and FN (red). Each image is a single 1$\mu$m z-slice acquired using a confocal microscope. Scale bar=10$\mu$m. (B) Bar graph quantifying the mean % colocalization between GM130 and FN on a single z-slice. % colocalization was determined using the coloc plugin on ImageJ. ROIs from more than 80 cells in each condition were included in the analysis. Statistical significance was determined using the unpaired t-test.

Full blots

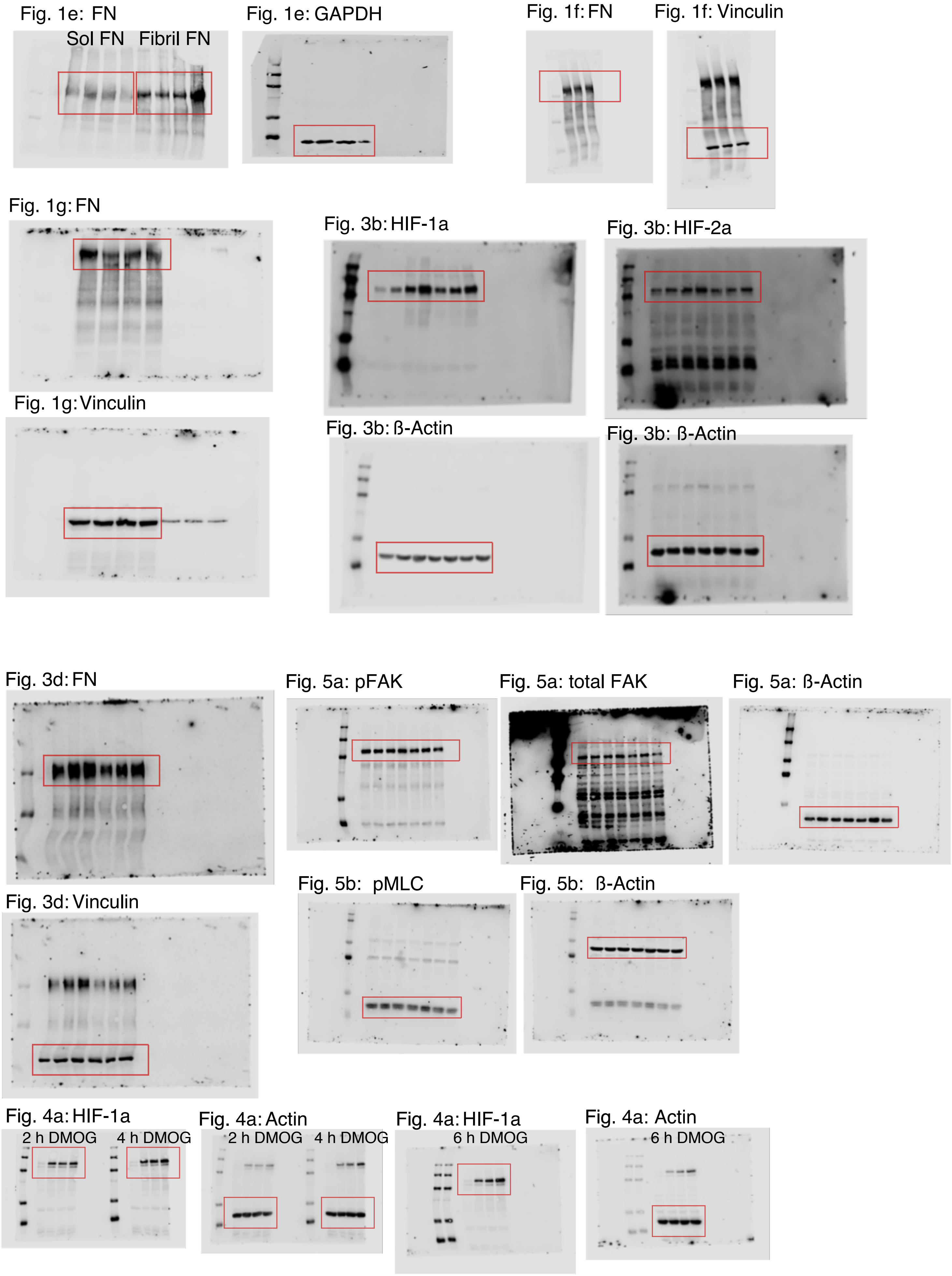

Fig. 1e: FN
Sol FN
Fibril FN
Fig. 1e: GAPDH
Fig. 1f: FN
Fig. 1f: Vinculin
Fig. 1g: FN
Fig. 1g: Vinculin
Fig. 3b: HIF-1a
Fig. 3b: HIF-2a
Fig. 3b: ß-Actin
Fig. 3b: ß-Actin
Fig. 3d: FN
Fig. 3d: Vinculin
Fig. 5a: pFAK
Fig. 5a: total FAK
Fig. 5a: ß-Actin
Fig. 5b: pMLC
Fig. 5b: ß-Actin
Fig. 4a: HIF-1a
2 h DMOG
4 h DMOG
Fig. 4a: Actin
2 h DMOG
4 h DMOG
Fig. 4a: HIF-1a
6 h DMOG
Fig. 4a: Actin
6 h DMOG

Full blots

## Supplementary Fig. S3 (A)

HIF-1a

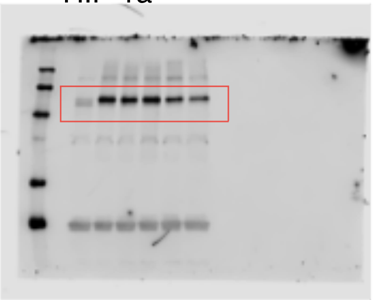

ß-Actin

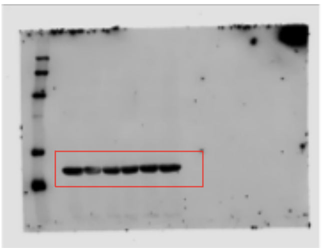

## Supplementary Fig. S3 (B)

HIF-2a

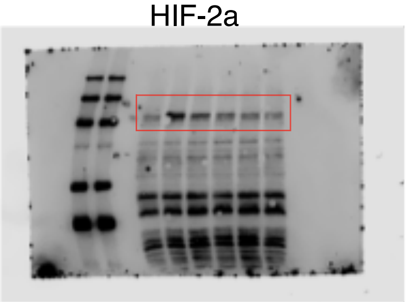

ß-Tubulin

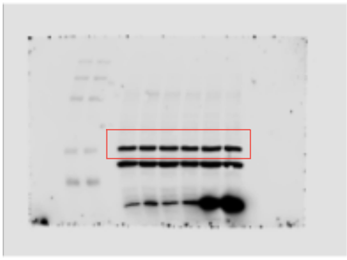

## Supplementary Fig. S5

pAkt(Ser473)

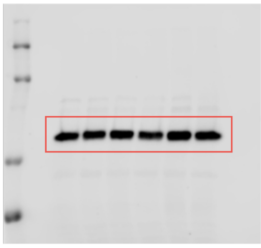

Akt

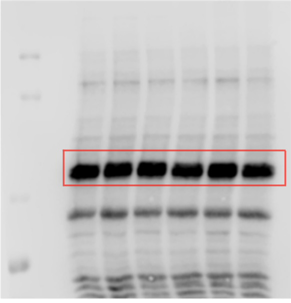

Tubulin

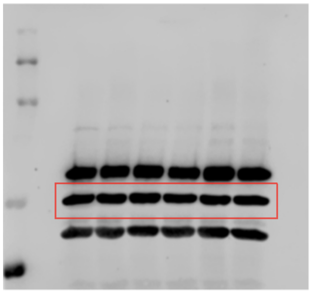

pAkt(Thr308)

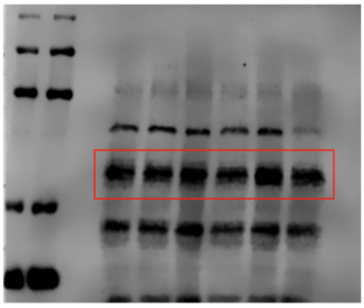

Akt

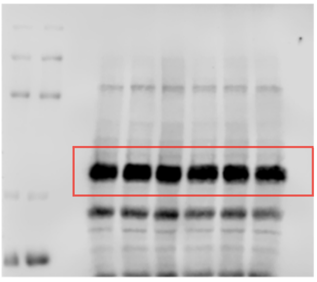

Tubulin

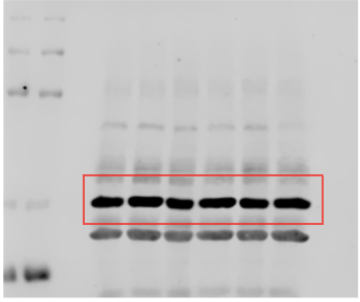

## Supplementary Fig. S7

PHD2

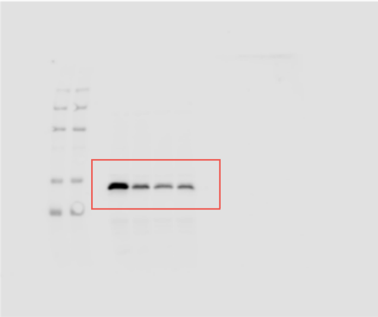

HIF-1a

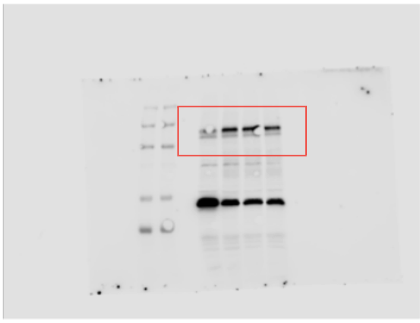

ß-Actin

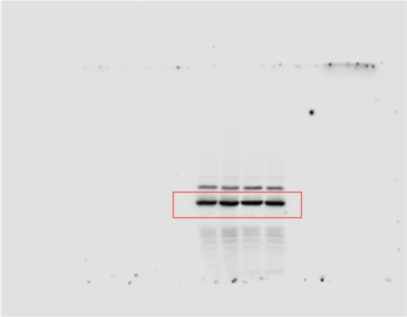

## Supplementary Fig. S9 (A)

alpha 4

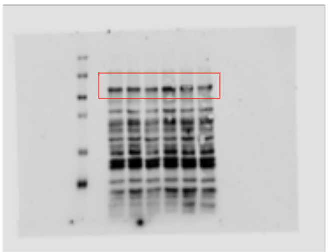

ß-Actin

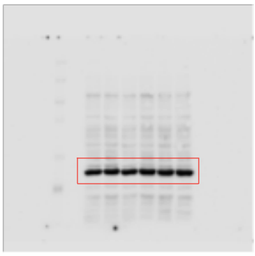

## Supplementary Fig. S9 (B)

Alpha 5

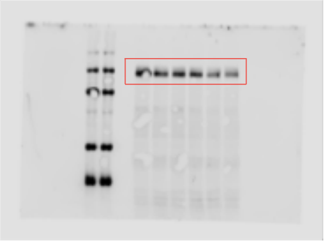

ß-Tubulin

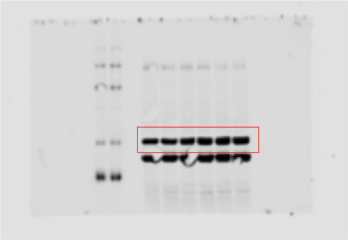

Supplement: Supplementary file 1 — Supplementary Figures [file 41598_2020_75756_MOESM1_ESM.pdf]
